# Causes of venous thrombosis

**DOI:** 10.1186/s12959-016-0108-y

**Published:** 2016-10-04

**Authors:** F. R. Rosendaal

**Affiliations:** Department of Clinical Epidemiology, Leiden University Medical Center, C7-P, P.O. Box 9600, 2300 RC Leiden, The Netherlands

## Abstract

Venous thrombosis which mainly manifests as deep vein thrombosis of the leg or pulmonary embolism occurs in 1 per 1000 per year. It occurs due to interacting genetic, environmental and behavioral risk factors. The strongest risk factors are certain types of surgery and malignancies. Over the last decade many new risk factors for venous thrombosis have been identified. Venous thrombosis has a high recurrence rate, of around 5 % per year. Whereas clinically it would be most important to identify patients at risk of recurrence, only male sex and a previous unprovoked thrombosis are established determinants of recurrent thrombosis.

## Background

Venous thrombosis, manifesting mainly as deep vein thrombosis of the lower extremities and pulmonary embolism, is still underresearched and underestimated. The World Health Organization lists the global impact of a large number of diseases in its Global Burden of Diseases, Injuries and Risk factors (GBD) publications, but does not include venous thrombosis. A recent study emphasised the lack of data for many regions of the world [1]. It provided data showing that annually over half a million deaths in Euorpe, and over 300 000 in the USA, can be attributed to venous thrombosis. It also showed that venous thrombosis was the leading cause of loss of ‘disability-adjusted life years’ (DALYs) in low and medium income countries, and the second leading cause in high income countries, with premature death as the main contributor. Public awareness of thrombosis is low, and fewer people have basic knowledge of its symptoms and risk factors than they have of other major vascular diseases as myocardial infarction and stroke [2]. To reduce the burden of thrombosis, it is necessary to know the factors that contribute to its occurrence. This will lead to risk stratification and targetted prevention and treatment.

### General epidemiology

#### Venous thrombosis by age and sex

**Fig. 1:**
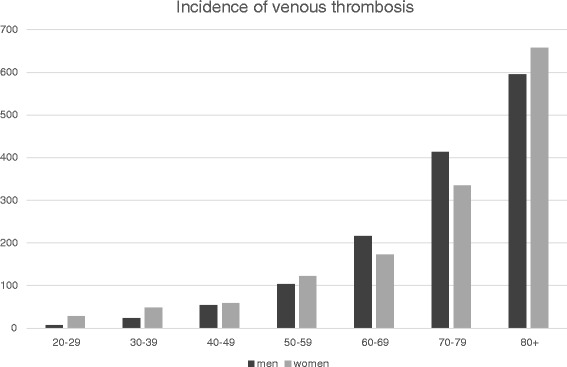
Incidence of venous thrombosis per 100 000 per year

Deep vein thrombosis and pulmonary embolism occur in 1–2 per 1000 individuals per year. There is a steep age-gradient, which follows a rule of 10: the annual incidence is 1: 100 000 in children, 1:10 000 in reproductive age, 1:1000 in later middle age and 1:100 in the very old [3]. The Tromsø study, in which the whole population of this Nordic city was followed over time, reported an overall incidence of first venous thrombosis of 1.4 per 1000 per year, with a slightly higher rate in women (1.6/1000 persons-years (py)) than in men (1.3/1000py) [3]. As Fig. 1 shows, the incidence is higher in women up till age 60, and then becomes slightly higher in men than in women. Venous thrombosis is a severe disorder with a poor prognosis, with an early case fatality rate of six percent, while 20 % of patients die within a year [3]. This is in part due to the relation between cancer and thrombosis, but even when patients with malignancies are excluded, over 10 % of patients die within the year.

### Ethnic differences

Several studies have shown a lower incidence in Asian than in European populations, with recent data from national health insurance claim data in Taiwan showing an incidence of 16.5 per 100 000 per year, i.e., a ten-fold lower incidence than reported from Europe and the USA [4]. Remarkably, the case fatality rate was similar if not higher than in the studies from Europe. Because many factors, including those related to the health care system, may cause differences in disease incidence and outcome between different geographic locations, studies that investigate individuals with different ethnic backgrounds living in the same country are highly interesting. One such study, from California, found slightly higher rates of venous thrombosis in blacks than in whites, and substantially lower (~5-fold reduction) in Asians [5]. A similar study from New Zealand corroborated these results, with a four-fold lower incidence of venous thrombosis in Asians than in those from European descent [6]. These data are compelling, but still there is some debate. Firstly, several studies have shown an equal rate of thrombosis after major risk factors such as surgery [7, 8], although a recent study from Taiwan showed a low risk of thrombosis after knee arthroplasty, and therefore the authors concluded that thromboprophylactic regimens could be less aggressive in Asians than in Europeans [9]. A report from Singapore showed that differences in medical practice do play a role, too: in 12 years time the proportion of patients with venous thrombosis per 10 000 hospital admissions had increased from 2.8 to 15.8 [10]. Although in all likelihood the incidence in Asians is lower than in Europeans, it should be borne in mind that this is unlikely to be the same everywhere and for every ethnic group in Asia. Moreover, risk factors for thrombosis seem to be the same in Asians as in Europeans, with the notable exception of factor V Leiden and prothrombin 20210A which are very rare in Asia. The absence of these two risk factors cannot explain the difference in incidences, since together they explain about 15–20 % of all thrombotic events in Europeans. The reason for the different rates between Asians and Europeans is unknown, although the persistence of the difference in ethnic groups in the United States and New-Zealand suggests a genetic cause.

The causes of venous thrombosis are genetic, acquired, behavioral and combinations of disease, and will be briefly discussed below. Table 1 list the main risk factors for venous thrombosis. Generally, and according to Virchow, risk factors can be related to stasis, hypercoagulability and changes in the vessel wall, of which the last category is more a risk factor for arterial disease than for venous thrombosis.

**Table 1:** Causes of venous thrombosis

Acquired			Genetic	Mixed
Medical	Drugs	Behavioural		
Age	Oral contraceptives	Obesity	Non-0 blood group	High FVIII
Major surgery	Postmenopausal hormones	Long-haul travel	Antithrombin deficiency	High VWF
Neurosurgery	In vitro fertilization	Smoking	Protein C deficiency	High FIX
Orthopaedic surgery	Chemotherapy	No alcohol	Protein S deficiency	High FXI
Prostatectomy	Psychotropic drugs	No exercise	Factor V Leiden (rs6025)	High prothrombin
Malignancy	Thalidomide	Exercise (elderly)	Prothrombin 20210A (rs1799963)	dysfibrinogenaemia
Trauma	Corticosteroids	Coffee	fibrinogen 10034 T (rs2066865)	Low TFPI
Prolonged bed rest		Strenuous work (arm thrombosis)	factor XIII val34leu (rs5985)	High PCI
Central venous catheter			SERPINC1 (rs2227589)	High fibrinogen
Plaster cast			FXI (rs2289252)	High TAFI
Myeoloproliferative disease			FXI (rs2036914)	Hypofibrinolysis
Heparin induced Thrombocytopenia			GP6 (rs1613662)	Hyperhomocysteinaemia
Hyperthyroid disease			FV (rs4524)	Hypercysteinaemia
Cushing syndrome			HIVEP1 (rs169713)	
Arthroscopy			TSPAN15 (rs78707713)	
Lupus anticoagulant			SLC44A2 (rs2288904)	
			ORM1 (rs150611042)	

*F* factor, *VWF* von Willebrand factor, *GP6* glycoprotein VI Platelet, *TFPI* tissue factor pathway inhibitor, *TAFI* thrombin activatable fibrinolysis inhibitor, *PCI* protein C inihibitor

### Genetic risk factors

The most prominent genetic risk factors for venous thrombosis are deficiencies of the natural anticoagulants protein C, protein S and antithrombin (reviewed in [11, 12]. These are quite rare in the general population (1:500 to 1:5000) and in family studies strong risk factors that increase risk 10-fold or more. Because they are so rare they are not important on a population level, and screening for them is not cost-effective, nor has a positive risk-benefit ratio been proven. While traditionally these were seen as the strongest risk factors for thrombosis, recently some doubt has been shed on that idea. Analyses in a large population-based study showed only moderate effects, in the order of a doubling of the risk [13, 14]. The reason for this huge difference is not clear, but may firstly be dilution: in the population studies deficiencies are based on phenotypic single measurements, and not on genotyping, so possibly true deficiencies are more rare and more severe. The most likely explanation, however, is that in families several abnormalities cosegregate and have synergistic effects [15]. A second group consists of genetic variants that increase the risk 2- to 5-fold. These include non-0 blood group, factor V Leiden, prothrombin 20210A and fibrinogen gamma’ 10034T (reviewed in [16]). These variants are present in several percent of the population. The first four are gain of function mutations, increasing factor VIII (non-0 blood group) or prothrombin (20210A), or making factor Va resistance to inactivation by activated protein C (APC-resistance). Gain of function mutations have an extremely low mutation frequency (since only in the most rare circumstance will a random mutation in an exon improves its function), which explains why factor V Leiden and prothrombin 20210A are not seen in Africans and Asians.

The third group are single nucleotide variants that are common and increase the risk of thrombosis by at most 50 % (reviewed in [17]. These include variants in coagulation factors, such as the antithrombin gene (*SERPINC1*), *F11 gene*, Glycoprotein 6 gene, but also several of unknown function, such as *HIVEP1*, *TSPAN15*, *SLC44A2*, *ORM1*. Obviously, risk factors that increase the risk of a first thrombosis by 50 % or less have no clinical relevance. However, when seen as a group, they may be relevant, as we observed a 100-fold gradient in risk when we looked at the aggregate effect of 37 genetic variants [18]. Moreover, the identification of genetic variants in genes not traditionally associated with coagulation may offer new targets for prevention and treatment, and possibly of antithrombotic treatment without a risk of bleeding.

### Acquired risk factors

As Table 1 shows, acquired risk factors can be associated with disease or surgery, with drugs and with behavior. Of all medical conditions, cancer is by far the strongest risk factor, increasing the risk of thrombosis over 50-fold in the first six months after diagnosis [19]. Notably, patients with cancer who develop thrombosis have a sharply reduced life span compared with cancer patients without thrombosis. Also very high risks are seen during surgery, in particular orthopaedic surgery and neurosurgery, which is why guidelines recommend thromboprophylaxis for all major surgeries and all orthopaedic interventions. Elevated levels of coagulation factors increase the risk of thrombosis, where the strongest effect is exerted by high levels of factor VIII.

Patients who are admitted to hospital have an increased risk of thrombosis, whatever the reason for admission. Traditionally, this has been attributed to immobilisation, but in the absence of healthy people being prescribed bed rest, it is difficult to decide whether it is the illness, possibly with inflammatory aspects, or the immobilization. In the one instance where healthy people were confined to bed for several months, in the setting of an experiment to study effects similar to lack of gravity with space travel, performed by the European Space Agency, we did not find any indication of clotting activation or clot formation [20]. Many drugs increase the risk of thrombosis, of which the most researched are female hormones, in oral contraceptives and in postmenopausal replacement therapy. Combined oral contraceptives, containing an oestrogen and a progestin increase the risk of venous thrombosis 2- to 4-fold (reviewed in [21]). In absolute terms the risk increase is small, since it affects women in reproductive age, whose baseline incidence is around 1 per 10 000 per year. Nevertheless, fatal thrombosis in a young woman is a tragedy, and therefore there is a moral imperative to search for the safest oral contraceptive. During the last few decades it has become clear that not only the oestrogen content but also the progestogen content affects the risk, and that oral contraceptives containing so called third and fourth generation progestogens (desogestrel, gestodene, drospirenone, cyproterone) confer a higher risk [21, 22].

The prevalence of obesity is increasing worldwide, and it is a clear risk factor for thrombosis, more than doubling risk for the obese [23]. Other life style factors have at most weak effects, of which the protective effect of moderate alcohol intake is noteworthy [24].

### Provoked and unprovoked thrombosis

Despite a large increase in the number of identified risk factors, still a substantial number of thrombotic events occur spontaneously. It seems likely that these simply reflect causes not yet discovered, maybe particularly genetic ones. The risk of recurrence is clearly higher after a first unprovoked than after a first provoed venous thrombotic event [25]. It seems the distinction between provoked and unprovoked thrombosis is not immediately relevant itself, but what is crucial is the difference between transient and persistent risk factors. Whereas few of the risk factors for a first thrombosis have a major role in predicting recurrent thrombosis, this risk is clearly very low after removal of a transient risk factor, such as surgery or oral contraceptive use [25, 26].

### Two paradoxes

Two paradoxes in venous thrombosis are what has been called the ‘factor V Leiden paradox’, and the observation that the incidence of first thrombosis is higher in women, while recurrent thrombosis occurs more often in men [27]. The factor V Leiden paradox is the observation that this genetic variant increases the risk of deep vein thrombosis but not of pulmonary embolism [28]. A series of possible explanations has been studied, but so far no explanation has been found [29]. This observation is highly relevant, for it implies that deep vein thrombosis and pulmonary embolism, for over 150 years seen as manifestations of one disease after the seminal work of Virchow, are different and may have specific risk factors [30]. The second paradox has been solved: when we, in a model, remove female-specific risk factors such as hormone use, pregnancy and puerperium, the incidence of venous thrombosis is also higher in men than in women [31]. The explanation for this difference, however, is still unexplained.

## Conclusion

Venous thrombosis is a serious disorder with a high incidence and a high burden of disease. The incidence varies between different ethnic groups, but underdiagnosis still plays a role which is related to poor public and even professional knowledge of the disease. The causes of thrombosis are both genetic and acquired and invariably both will play a role. The strongest risk factors are cancer and certain types of surgery. Risk factors of intermediate strength are life style factors such as hormone use and overweight, as well as genetic factors such as deficiencies of natural anticoagulants. Many other genetic risk factors have been identified that are common, but only have a weak effect on thrombosis. Future aims to come to targeted prevention and treatment will follow from the integration of the large number of risk factors in individual risk prediction tools.
